# Endothelin-based markers for endothelial dysfunction in chemotherapy-induced cardiotoxicity

**DOI:** 10.1016/j.jmccpl.2023.100053

**Published:** 2023-10-13

**Authors:** Gabrielle Boutin, Jale Yuzugulen, Md Zahidul Islam Pranjol

**Affiliations:** a School of Life Sciences, University of Sussex, Brighton, UK; b Faculty of Pharmacy, Eastern Mediterranean University, Famagusta, North Cyprus via Mersin 10, Turkey

**Keywords:** Cardiotoxicity, Biomarkers, Endothelin-like domain peptide, Endothelial dysfunction, Endothelin-1

## Abstract

Current cardiac biomarkers, troponins and brain natriuretic peptide, are primarily used to assist in the diagnosis or exclusion of myocardial damage and congestive heart failure, respectively. The use of these biomarkers in chemotherapy-induced cardiotoxicity has been evaluated by various studies. However, neither biomarker provides early predictive value, leaving many cancer survivors with irreversible cardiac injury. Assessing endothelial dysfunction could be an effective measure of chemotherapy-induced cardiotoxicity at the vascular level. Risk profiling and detection of vascular toxicities may offer predictive biomarkers to prevent chronic manifestation of irreversible cardiotoxicities. Emerging interest has developed in finding biomarkers that could ideally provide earlier prognostic value. Thus, the aim of this review is to give an overview of current blood-based cardiac biomarkers and discuss the potential of endothelin-1 (ET-1) and more stable peptide fragments of ET-1 synthesis as biomarkers of endothelial dysfunction. For instance, endothelin-like domain peptide (ELDP) and C-terminal pro-endothelin-1 (CT-proET-1) demonstrated high-sensitivity and longer clearance rate than ET-1. Thus, investigating their biomarker role in chemotherapy-induced cardiotoxicity is important and could provide additional insights for identifying patients at risk. Also, additional research is required to fully understand ELDP-mediated vasoconstriction. This review will discuss the future development of ET-1, ELDP and CT-proET-1 as prospective predictive biomarkers.

## 1 Introduction

Development of chemotherapy has improved treatments against malignancies, significantly increasing survival rate [[1], [2], [3]]. Chemotherapeutics such as anthracyclines, alkylating agents, antimetabolites and taxanes efficaciously treat malignancies in adults and children [4]; albeit accompanied by adverse effects. As such, chemotherapy-induced cardiotoxicity (CIC) is prevalent among cancer survivors [3,4]. Severity of CIC is associated with late detection, dense doses, and underlying comorbidities [[5], [6], [7]]. Onset symptoms typically manifest after treatment has completed [8]. Acute-onset incidences could be reversed if detected early [6]. The two classifications, early- and late-onset, are irreversible with poor prognosis and limited treatment options [3,6].

A 9-year surveillance study of 63,566 breast cancer cases reported that cardiovascular disease (CVD) was the leading cause of death among all women (15.9 %), followed by breast cancer (15.9 %), revealing CIC as a greater risk than cancer diagnosis [1,3,5,7]. Early detection of CIC is essential to prevent toxicity and improve patient wellbeing. Current research predominantly focuses on cardiac pathophysiology and overlooks vasculature influences [8,9]. Cardiac biomarkers, troponin (cTnI), and brain natriuretic peptides are presently utilised as markers for cardiotoxicity during chemotherapy (e.g. doxorubicin) treatment [2,10]. These cardiac markers do hold limitations as they only indicate myocardial damage, failing to predict CIC with regards to vascular damage [2,11]. For instance, the administration mode of many chemotherapies is intravenous infusion [12], with vascular endothelial lining at the initial contact, potentially resulting in endothelial damage [8]. The endothelium mediates systemic functions with controlled release of vasopressors, endothelin-1, angiotensin II, and nitric oxide (NO) [13,14]. Exposure to chemotherapy induces dysfunction of the master vascular regulator, also known as endothelial dysfunction (ED), through disruption upon perfusion, coagulation, vascular tone, and thrombolysis [[15], [16], [17]].

ED has become an emerging factor in CIC. [[17], [18], [19]]. Data has highlighted the correlation of disease progression with increased plasma concentrations of systemic biomarkers [1,8,11,15]. Endothelin-1 (ET-1) has been associated with the progression of systemic illness related to the pulmonary and renal systems [16,20,21]. Cardiac diseases have also displayed similar observations [[2], [3], [4],[7], [8], [9],^11^,^15^,^17^,^18^]. This review will discuss the roles of ET-1 and co-released peptides in cardiotoxicity.

## 2 Chemotherapy-induced cardiotoxicity

### 2.1 Acute cardiotoxicity

Injury from chemotherapy is evident upon the cardiac system, although toxicity varies based on the dose and regime of chemotherapy [[22], [23], [24], [25]]. Different classifications include acute, early-onset and late-onset chronic progression [3,26]. Type 1 irreversible damage is often acquired from high-cumulative dose, with type 2 categorised as reversible and often not directly attained from high-load [26,27].

Acute-onset (type 2) follows a single dose or course, with symptoms present within 14 days of treatment [6,28]. Each classification of chemotherapy possesses risk to develop cardiotoxicity [28,29]. Such complications include arrhythmias, myocardial infarction, ischemia, vasospasm, thromboembolism, atherosclerosis, hypertension (HTN), heart failure (HF), and reduced left-ventricular-ejection-fraction (LVEF) [[28], [29], [30], [31], [32], [33], [34]]. Chemotherapeutic agent such as cyclophosphamide acts by inhibiting protein synthesis and cross-linking of nucleic-acid strands, with selective suppression among regulatory T-cells [34,35]. However, this could cause adverse effects. For instance, a meta-analysis of cyclophosphamide courses found 7–28 % of participants had reduced LVEF within 10 days of initial administration, with HF correlation against dosage [33]. Another study suggested that at 5 % of cyclophosphamide, patients suffer immediate cardiac-related issues [3].

Reduction of single high-load doses via dual treatment has shown to decrease severity of acute adverse effects [36]. However, oxidative stress and subsequent cardiomyocyte death remain a major cause for acute cardiotoxicity [37]. Risk profiling for acute cardiovascular complications have improved, albeit chronic-CIC still remains critical [38].

### 2.2 Chronic cardiotoxicity

Chronic cardiotoxicity falls under type 1 classification with early- and late-onset diagnosis [39]. Early-onset progresses within 1 year with diseased cardiomyocyte forecasting HF [40].

Late-onset complications can arise many years after treatment, often attributed to the dosage and associated with a less favourable prognosis [6].

Most potent of chemotherapeutics inducing chronic cardiotoxicity are cytotoxic-antibiotics, anthracyclines [40]. As such, cardiac dysfunction is observed nearly immediately following initial dose administration [41], with prospective damage to dilated hypokinetic cardiomyopathy, non-obstructive and obstructive coronary artery disease, and HF [40]. Anthracyclines intercalate by inserting a planar tetracyclic chromophore between the base pairs of nucleic acids, thereby halting the progression of the cell cycle [42]. This could also alter mitochondrial function via topo-isomerase 2 inhibition and generate free radical oxidative stress [43]. Such cardiovascular impairments develop slowly with type 1 myocardial and left-ventricular damage [44].

A 2021 cohort-study on 10,209 female breast cancer patients during 2000–2009 reported that 3.39 % of anthracycline patients developed HF with dose dependent correlation and 29.92 % of patients with combined anthracycline-trastuzumab sustained HF within 2 years post treatment [45]. A total of 6.8 % of patients experienced chronic HF for a duration of up to a decade after treatment, and among them, one-third experienced notably severe symptoms [45]. In a subsequent cohort study conducted in 2007, where 12,500 women diagnosed with invasive breast cancer were included over an 8-year period, similar findings were observed [36].

It was determined that patients who did not undergo chemotherapy had a significantly lower risk of HF compared to those who received anthracycline treatment. Specifically, 95 % of the patients treated with anthracycline experienced HF or cardiomyopathy [36]. Combining both anthracycline and trastuzumab treatments led to reduced comorbidity, primarily among younger patients who were in a lower-risk category [36].

Monoclonal antibody-based trastuzumab (also known as Herceptin) has been shown to induce chronic cardiotoxicity. For instance, long-term administration has been reported to indefinitely damage cardiomyocytes and left-ventricular wall [46]. Thus, chronic cardiotoxicity can decrease 50 % LVEF 5-years after treatment, with only 11 % patients recovering fully [40].

The beneficial effects of trastuzumab treatment have shown consistent results regardless of the duration of the treatment regimen, while the occurrence of substantial cardiotoxicity remains a notable concern. An 11-year follow-up study involving 9901 breast cancer patients yielded results indicating that a 2-year trastuzumab treatment course did not confer significant benefits over a 1-year course [47], indicating potential for reduced duration for treatment administration. The same study also showed a 2-fold increased risk of late-onset HF from trastuzumab than chemotherapy alone [47]. Additionally, lifestyle and epigenetic factors have been described as contributing risk factors [48,49]. Studies that examined Danish, American, and English populations found a reduced occurrence of HF following trastuzumab treatment among specific subsets of individuals adopting healthy lifestyle choices [50]. Regardless of behaviour, cardiotoxic effects persisted across all demographic groups.

Despite known induced cardiotoxicity, anthracyclines, trastuzumab and other antineoplastics are highly effective in the treatment of cancers [51]. While innovative approaches aimed at reducing cardiotoxicity have been developed for low-risk cases, such as liposomal encapsulation [52,53], individuals classified as high-risk continue to exhibit increased susceptibility to develop HF due to cardiomyocyte injury and a reduction in LVEF [54,55]. Early detection of cardiotoxicity risk through biomarker measurements provides an opportunity to intervene and avoid irreversible damage [56,57]. Active involvement in cancer management is needed with the use of personalised approaches in therapy and cardioprotective interventions when necessary [43,58]. Assessing cardiac biomarkers throughout treatment could predict the switch from acute type-2 features to chronic type-1, including ED [59]. Therefore, early intervention could potentially prevent CIC and improve survival rate [60].

## 3 Endothelial dysfunction

Cardiotoxicities such as reduced LVEF and hypertrophy, remain major concerns [61]. However, the key regulator of the vascular physiology, endothelial cells, remains at risk of damage by administration of chemotherapies. Once administered, chemotherapeutic agents interact with endothelial cells (ECs) on the luminal surface of blood vessels, making ECs highly vulnerable and resulting in a disruption of the endothelial physiology and dysfunction.

**Fig. 1:**
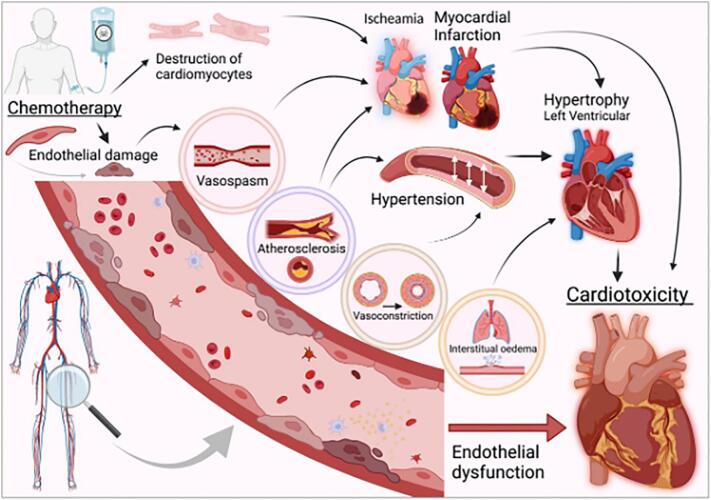
Series of adverse effects from chemotherapeutics causing endothelial dysfunction and associated cardiotoxicity.

ED is defined by enhanced vasoconstriction, inflammation, and thrombolytic events [62]. With induced vascular toxicities [15], ED systemically proceeds towards non-coronary artery disease, HF and CIC [63]. Fig. 1 illustrates chemotherapy-induced ED and cardiac complications.

It is well known that chemotherapy progressively damages cardiovascular health [26]. However, disruption of the physiological endothelial health is believed to be a key contributor of the overall CIC. ED could be caused by chemotherapy-induced reactive oxygen species production that effects perfusion, reduces NO bioavailability, and progresses atherosclerosis [19]. Unhealthy lifestyle choices and epigenetic factors also contribute to a decrease in endothelial function, primarily through the generation of reactive oxygen species [[62], [63], [64], [65]].

Venous thromboembolism had remained a clinical focus until arterial vasculature complications were identified [15]. Recent focus has highlighted that arterial toxicities have a direct adverse impact on cardiac function. When the endothelium's integrity is disrupted, there is a clear progression towards chronic vasoconstriction, ischaemia, hypertrophy, hypertension (HTN), and vasospasm [64,[66], [67], [68], [69]]. Pulmonary and renal complications such as diffuse alveolar haemorrhage and glomerulonephritis respectively, are frequently observed in association with CIC [19,62,64,70]. The physiological consequences resulting from ED are dependent upon the metabolic rate of the specific organ, the degree of exposure to ED, and the duration of the vessel abnormality [71]. Under resting condition, the cardiac tissues preserve oxygen to maintain sufficient perfusion. This renders the myocardium particularly vulnerable due to its elevated metabolic rate. Furthermore, arterial response to hypoxia and increased metabolic demand is diminished by ED through reduced vasodilation [19].

Severity of ED has been shown to correlate with measure of cumulative chemotherapy dose and category [72]. High dose of chemotherapy disrupt endothelial function with impaired vascular tone, reduced platelet regulation and innate immune response [64,65,73]. Peripheral resistance and activation of vascular inflammation trigger endothelial-derived vascoactive factors and results in increased vascular and sympathetic tone [71,74]. Short episodes of chemotherapy-induced ED can often be reversed with permissive risk-reduced therapy [60,75]. Irrespective of lesion regression, recovery is achievable with prompt early detection of ED [62].

## 4 Current blood-based cardiac biomarkers

Chemotherapy-induced cardiotoxicity is currently monitored by utilising two serum biomarkers, cardiac troponin and N-terminal pro-brain natriuretic peptide, which have shown prognostic utility of cardiac injury.

### 4.1 Cardiac troponin

Myocardial damage is detected by reduced plasma concentrations of cardiac troponin isoforms I and T, (cTnI) and (cTnT) and is used to diagnose acute coronary syndrome [[76], [77], [78]]. Myocardial infarction (MI) and reduced LVEF are frequently affiliated with stimulated cardiac troponin release following anthracycline treatment [79,80]. Lakhani et al. studied 17 newly diagnosed females with triple-negative (ER-, PR-,HER2-) invasive ductal carcinomas with assessments at 3 and 6 months after anthracycline administration, where a substantial LVEF reduction at 6 months was observed [81]. A study by Ky et al. examined cardiopathy across 78 patients with HER2+ breast cancer with prescribed doxorubicin-trastuzumab regime where a dose-dependent correlation was observed between cTnI and acute-cardiotoxicity [82]. Doxorubicin-induced cardiotoxicity shows a greater risk when the dosage is increased, particularly among younger patients [79], with progressive cardiotoxicity and HF effecting 4.8 % of survivors by the age 45 years [83]. A meta-analysis of 19 studies also demonstrated increased troponin levels across 1182 subjects and 5 cancers, yet detection within 3 months of initial therapy demonstrated unaffected troponin levels [83]. A large study encompassing 703 cancer patients, evaluated the potential of serial monitoring of cTnI at multiple intervals across chemotherapy treatments; the highest incidence of cardiotoxicity was observed in patients with elevated cTnI within 72 h of chemotherapy, which persisted one month post treatment [84]. Cardiac troponins levels correspond to coronary impairment and present late-stage detection [85]. However, confirming the specific effects induced by chemotherapy remains challenging, as there is a risk of unrelated troponin release [79,[81], [82], [83],^86^]. Combined approaches of monitoring with cardiac biomarkers and imaging modalities, including echocardiographic variables, enhance risk profiling [87]. Still, use of cardiac troponins as independent predictive biomarkers in CIC is subsequently minimal. Biomarkers which provide greater prediction include analysis of peptides relevant for systemic integrity, over loss of cardiac muscle.

### 4.2 Natriuretic peptides: brain natriuretic peptide and its N-terminal fragment

The neurohormone brain natriuretic peptide (BNP) is produced and released by left ventricular cardiomyocytes following induced stress in the walls [88]. BNP and its biologically inactive precursor N-terminal pro-BNP (NT-proBNP) have both shown clinical application for HF, with possibility for early detection [[89], [90], [91], [92]]. However, results from BNP studies show opposing results. A meta-analysis by Rüger et al. compared NT-proBNP serum levels from 853 patients with early-stage breast cancer, treated with various high-load chemotherapies over 18 weeks [93]. Cardiotoxicity was detected in 12.9 % of patients during therapy; NT-proBNP concentrations quickly upsurged, with significant association at 6-weeks with a secondary amplified fraction of NT-proBNP concentrations towards final weeks [93]. Lenihan et al. investigated BNP values 24 h after anthracycline-cycle from 109 subjects, where 10.1 % patients suffered from cardiac events, including symptomatic HF and sudden death, with consistent BNP levels >100 pg/mL pre-incident [94]. An extensive childhood-cancer meta-analysis conducted from 2001 to 2015 observed elevated BNP and NT-proBNP concentrations during and post treatment [83]. The review reported significant sensitivity towards acute-CIC, over chronic-onset of childhood survivors with higher incidence of LVEF dysfunction upon elevated BNP-associated concentrations [83]. BNP and NT-proBNP, which are effective for assessing incidents, can be affected by various other responses at the same time [75,90,91] and require additional assessments for diagnostic accuracy [93]. NT-proBNP is recognised to provide greater sensitivity for prognostic detection [95], though offers little value for predictive purposes and fails to monitor vascular health.

### 4.3 Detection methodologies of current cardiac biomarkers

Cardiac troponins can be accurately measured a few hours after incident [96]. Levels peak between 24 and 48 h and can remain in a patient's circulatory system a week post cardiac arrest [97]. Although extremely specific and accurate, cardiac troponins remain useful for detection of injury post incident, absent of any predictive value.

Goryacheva et al. summarised detection methodologies for BNP and its peptide fragment NT-proBNP in HF [98]. In general, chemiluminescence based immunoassay methodology is used for measuring BNP/NT-proBNP [99]. BNP levels lower than 100 pg/mL rules out acute HF risk [100]. When tested, BNP levels were observed to vary in different ethnic groups (summarised in Table 1) [101]. According to the regulatory guidelines, NT-proBNP levels >125 pg/mL is considered as a significant risk factor for HF, while 400 pg/mL is indicative of chronic HF [102,103]. Although NT-proBNP has demonstrated most favourable accuracy, there are still limitations in defining NT-proBNP ranges diagnostic of HF. For instance, patients of the same cardiac illness with darker skin were shown to have lower levels than lighter skinned patients [100]. Thus, although these biomarkers were shown to offer some predictive value across ethnicities [104], false positives are common due to their degree of sensitivity [105]. Additionally, the levels of NT-proBNP were shown to be higher with increasing age, underscoring the importance of establishing age- and gender-specific reference ranges [106,107].

**Table 1:** Summary of detection methodologies for cardiac troponins and NT-proBNP.

Biomarkers	Detection methodology	Healthy range values	References
Cardiac Troponin (T and I)	Chemiluminescence One step immunoassay, Digital immunoassay	cTnI <10–20 pg/mL	[108,109]
		cTnI >23 pg/mL and cTnT >20 pg/mL associated with HF	[100,110]
NT-proBNP	Triage Fluorescence immunoassay; Finger prick test method for rapid quantitative assays, applied clinically for emergency admissions Electro-chemiluminescence immunoassay Roche Cardiac proBNP + test uses classic lateral flow immunoassay principle with gold nanoparticles: Roche Diagnostics	**Black patients:** 10–52 pg/mL **Hispanic patients:** 14–59 pg/mL **White Caucasian patients:** 16–62 pg/mL	[98,100,101,111]
		Healthy ranges have not been exclusively determined due to irrelevant stimuli causing synthesis and plausible unrelated release.	[98,104]

Table 1 provides a summary of current detection methods used for cardiac troponins and NT-proBNP.

Table 2 summarises advantages and disadvantages of troponins and NT-proBNP in assessing cardiotoxicity and ED.

**Table 2:** Summary of advantages and disadvantages of current biomarkers to measure cardiotoxicity.

Current cardiotoxicity biomarkers	Advantages	Disadvantages	Source
Cardiac Troponin (cTnI and cTnT)	- Stable in circulation. - Reliably long half-life. - Understood role and respective mechanisms. - Directly released following damage to cardiomyocytes. - Wide range of clinical evidence to validate cardiac function in response to injury.	- Release from damage provides diagnostic values, but no predictive value for risk stratification. - Limited to cardiac injury, not expressly related to vasculature.	[[81], [82], [83]]
NT-proBNP	- Stable in circulation. - Synthesised and secreted in response to ventricular stress. - Elevations observed repeatedly with reduced LVEF. - Breadth of clinical research and accepted as a predictive biomarker by various guidelines to be monitored in HF - Understood role and respective mechanisms.	- Contradictory findings providing unreliable biomarker application (BNP). - Found to be hypersensitive and easily stimulated by gonad corticoids, and patients of diabetes mellitus and cancer, discrediting interpretation of results (NT-proBNP and BNP). - Significant values in plasma concentrations appear only after substantial damage has occurred (BNP). - Not viable as an independent marker (NT-proBNP and BNP) for diagnosis. - Alterations occur with differing age, gender, and ethnic groups.	[81,83,93,94]

The circulatory system and essential organs rely on endothelial function. Studying this system could be more advantageous for detection compared to analysing injured cardiac muscle. Importantly, assessing ED can predict potential risks.

## 5 Potential biomarkers for endothelial dysfunction

### 5.1 Endothelin-1

ET-1 plays a role in electrolyte clearance, central and peripheral sympathetic activity, blood pressure maintenance, alongside ionotropic and mitogenic influences [112]. ED disrupts the renin-angiotensin-aldosterone system and shifts the balance for overexpression of ET-1, while decreasing NO generation [113]. Failure to control systemic vasopressors is associated with progressive vascular diseases including cardiac, renal, and pulmonary HTN with secondary diseases such as acute and chronic HF. Accordingly, ET-1 has been implicated mechanistically in various CVDs [64,70,[113], [114], [115]].

Chronic activation of the compensatory neurohormonal system is known to produce haemodynamic pressure, with descending cardiovascular integrity. In addition to chronic heart and circulatory disease, acute and rapid onset HF resumes to demonstrate ET-1 as a progressive marker [115,116]. Dmour et al. reviewed association from elevated ET-1 concentrations with poor morphofunctional outputs, renal function, and high mortality rate from cardiotoxicities [115]. Clinical study by Zymliński et al. evaluated 284 acute HF patients across 48 h for ET-1 presence alongside organ dysfunction [116]. ET-1 detected upon admission served as an indicator for an unfavourable prognosis among patients with between none and three organ injuries (baseline ET-1 7.4 pg/mL, 8.8 pg/mL, 9.8 pg/mL, and 10.8 pg/mL, respectively) [116]. Greatest risk was observed among three organ injuries, provisioning 46 % survival in the first year [116]. More recently, in a clinical trial, higher baseline for ET-1 was found to be associated with worse clinical outcomes where a rapid decline in kidney function was observed [117]. Moreover, type-2 diabetic drug dapagliflozin induced a moderate reduction in serum ET-1 [117]. Thus, ET-1 has the potential to serve as a marker because its measurement may indicate an underlying problem. Further studies are still required to provide better understanding of factors affecting ET-1 levels including drug therapies.

In disease, ET-1 travels systemically, from site of trauma or signal response, towards sites of high metabolic rate. This could generate a high ionotropic effect upon cardiac muscle leading to a decrease in LVEF [64,118]. Krishnarao et al. evaluated breast tissue specimens from 33 female patients under anthracycline treatment [119]. Here, ET-1 expression was reported to correlate with reduced LVEF, with greater quantities allied to <10 % reduction in LVEF.

High plasma concentrations of big-ET-1, precursor protein of ET-1, have also indicated ED, and worse risk of HF [120]. A 2017 cohort study followed 3154 patients with stable coronary artery disease for 24 months. The results indicated an upsurge in the endothelin-derived peptide was associated with increased cardiovascular risk [121].

A strong association between ET-1 and cardiovascular toxicities has been established [118,119,121]. Plasma concentrations of ET-1 are not reliable measures due to varied effectors including immediate receptor uptake, unregulated secretion through albumin release at the basolateral cell surface, with an ambiguous clearance pathway [70,118,122,123]. Clinical application of big-ET-1 and ET-1 fails due to poor detection, as both share a short circulating half-life, which is 1 min for ET-1 [118,120,121,123,124]. Other preproET-1 peptides conversely have shown better stability in circulation up to 60 min [123]. Therefore, their potential as more stable peptide fragments of preproET-1 has been investigated with great interest.

### 5.2 Discovery of other PreproET-1 peptides: endothelin-like domain peptide and C-terminal proET-1

Endothelin-Like Domain Peptide (ELDP) was first identified in 1989 as co-secretory peptides, but their biological function remained poorly understood [125,126]. Novel interest has emerged where ET-1 precursor proteins ELDP and C-terminal-proET-1 are considered as potentially more reliable predictive markers than ET-1, with characteristics of greater sensitivity and correlative specification for cardiovascular pathologies [114,123].

PreproET-1 modification initiates intracellularly; within the endoplasmic reticulum, a signal peptidase cleaves 17 amino acid residues from the N-terminal of the peptide. Proteolytic processing at double basic amino acid residues with proprotein convertases (i.e. furin) leads to the generation of other proET-1 peptides. Cleavage by carboxypeptidases forms the inactive big-ET-1, which is then converted into ET-1 by endothelin-converting enzyme (ECE) [127]. Yuzugulen et al. characterised the proET-1 peptide fragments as CT-proET-1 (preproET-1: 169–212), NT-proET-1 (preproET-1: 18–50) and ELDP (preproET-1: 93–166); and showed ELDP specifically modulated ET-1 [123]. Their findings established CT-proET-1 and ELDP as potential plasma biomarkers for cardiovascular disease; with slow clearance rate, relative sensitivity, and specification prospects [123].

**Fig. 2:**
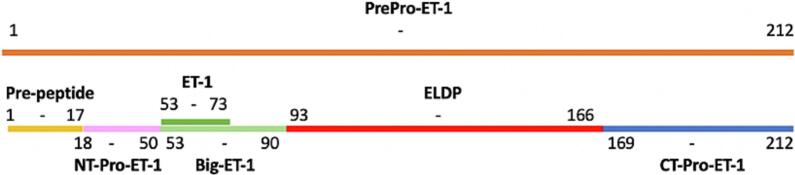
PreproET-1 peptide fragments with respective sequence order. Endothelin-like domain peptide (preproET-1[93–166]) and CT-proET-1 (preproET-1[169–212]).

ELDP resides within preproET-1, secreted during big-ET-1 processing. These additional endothelin-like peptide fragments are co-secreted with active ET-1 [123,128]. As shown in Fig. 2, the ELDP sequence resides within the precursor preproET-1, alongside other potential markers [114].

### 5.3 ELDP and CT-proET-1: pre-peptide and sensitivity analysis

Characterisation of these peptides revealed ELDP to display similar expression patterns to ET-1, during ex vivo precursor analysis of human aortic endothelial cells [123]. The levels of primary peptides involved in proET-1 processing were also confirmed after early passage of extracted cells [123].

Current endothelin-receptor antagonists are administered to halt progression of pulmonary HTN [58]. Risk factors and symptoms are successfully reduced, yet the endothelin-1 (EDN1) gene expression is not inhibited with endothelin-receptor antagonists [129]. The lack of efficacy from vascular treatments suggests that the endothelin systems are not fully understood. ET-1 associated peptides were until recently, considered non-functional [130].

From this study, the levels of ELDP closely resembled that of ET-1. It is possible to infer that the clearance rate of peptides is influenced by peptidases induced by tumor necrosis factor-alpha (TNF-α) [123]. Regarding ET-1 and ELDP, TNF-α displayed a decreased transcription efficacy compared to more sensitive markers e.g. CT-proET-1 [123]. Notably, all precursors exhibited positive R values in correlation with the expression of ET-1 (r = 0.86–0.93). Minor differences in gene expression were observed between the peptides. TNF-α stimulation induced EDN1 gene expression and subsequently in increased levels of NT-proET-1 and CT-proET-1. However, ET-1 and ELDP levels did not alter, indicating possible clearance of these peptides due to TNF-α-induced peptidase activity or receptor-mediated clearance. This may suggest that ELDP closely resembles ET-1 more than other co-synthesizers, making it a candidate with higher biomarker potential [123].

Clinical benefits of plasma biomarkers is characterised by, among other factors, a slow clearance rate to generate a longer detection period [131]. Yuzugulen et al. investigated elimination rates of pre-peptides through bolus-administration in anesthetised rats at the concentration of 1 nmol/kg [123]. Arterial blood samples were collected at 30 s, 1, 2, 5, 10, 20 and 40-minute post administration and analysed. In the investigation, plasma NT-proET-1 concentrations showed a significant reduction at 30 s, near complete removal by 5 min, with estimated half-life below 20 s. On the contrary, a significantly slower rate and two-phase mechanism was exhibited by CT-proET-1 and ELDP; half-life estimated at 42 and 30 s initial phase, followed by 7.3 min and 5.7 min in phase two [123]. Thus, CT-proET-1 held significant biomarker advantage over ELDP in respect to slower clearance, although both showed longer half-life than ET-1 [118,123]. These molecules exhibited potential as biomarkers, showing promise for their utility in human vascular diseases.

### 5.4 ELDP and CT-proET-1: cardiovascular and systemic diseases

Increased plasma concentrations of ELDP and CT-proET-1 were reported in vascular diseases, which were indicative of disease progression [70,123,132]. Development of chronic kidney disease (CKD) and decline of glomerular filtration rate are associated with increased ET-1 and proET-1 plasma concentrations [70,114,115,118,132].

Poor renal function has become a strong predictor for HF and cardiotoxicity [32,115,118]. The renal and cardiac systems communicate via regulation of fluid and electrolyte homeostasis by the kidneys, to prevent hypoperfusion and support efficient cardiac output [133]. ED diminishes kidney function, with deterioration symptoms equivalent of CIC [134]. Complications like venous blockage, retention of electrolytes, and strained blood vessels necessitate an increased effort from both the heart and kidneys to pump blood in response to systemic hypotension [135]. The haemodynamic regulations between these two systems propose utility as indirect markers, based on systemic function [133].

An earlier experiment conducted by Yuzugulen et al. demonstrated enhanced ELDP concentrations throughout progression of CKD stages [132]. Compared to control (6.34 + 1.4 pmol/L), early stages (1–2, 6.16 + 1.43 pmol/L and 7.01 + 2.15 pmol/L) CKD did not show any significant differences in ELDP concentrations. However, CKD stages 3–5 demonstrated significant elevations (7.74 + 1.64 pmol/L, 8.96 + 3.82 pmol/L, 12.35 + 4.45 pmol/L), and stage 5 covered near two-fold increase in ELDP levels compared to control. Urine samples were concurrently tested with serum, however, revealed no significant change in ELDP levels [132]. Dhaun et al. reported consistent findings, where ELDP levels were higher in more advanced stages of CKD [70]. The concentration pattern was reproduced and resembled previous findings with CKD stage 5 ELDP levels were double the control range [70]. Urine analysis again displayed no significant differences in ELDP levels. Detection of lower levels in urine could be indicative of alternative peptide processing in urine. Furthermore, the concentrations may fall below the detection range of the assays. To ensure the reproducibility of the findings, subject-related variables such as age, lifestyle, and gender were matched between the control and patient groups [70,132]. Plasma ELDP in both circumstances inversely correlated, with minor quantities present in control and early CKD.

The above provides evidence of ELDP as an indicative biomarker for deteriorated vascular systems. CT-proET-1 was investigated alongside; data correlated progression of CKD, with higher plasma concentrations than ELDP [70]. CT-proET-1 has been monitored in a much wider range of vascular conditions including infection induced-sepsis, and systemic and pulmonary HTN [[136], [137], [138]].

Pulmonary HTN is a systemic condition caused by persistent high blood pressure, affecting dexter-cardiac arteries and lungs [136]. Renovascular HTN affects arterial vessels in the kidney [139]. Most concerning, pulmonary arterial HTN that initiates pulmonary arteries to narrow until blocked, lesioned and destroyed [140]. Vascular stiffness, arterial stenosis and vessel shrinkage are common consequences of ED, associated with alkylating-agents and docetaxel [136,141]. HTN and other cardiovascular diseases share dysregulated and increased release of pro-peptides [70,114,132]. With higher ET-1 production, release of other more stable peptide fragments, CT-proET-1 and ELDP, are observed with predictor utility between mild-low risk and chronic-severe conditions [70,123].

Yuzugulen et al. assessed plasma concentrations of ET-1 pre-peptides as biomarkers for cardiovascular diseases, chronic HF matured from ischemic heart disease and a control group with mild HTN [123]. Observation of ELDP and CT-proET-1 were substantially larger in patients with chronic HF, with decreased levels for mild hypertensive controls [123]. Further analysis displayed ELDP and CT-proET-1 measures were consistent with expected levels for ET-1 production [123], further verification of co-synthesis; with CT-proET-1 showed higher concentrations, 80 % more than ELDP.

Collectively ELDP has sustained a presence across systemic and cardiovascular diseases, with potential use for risk stratification for patients undergoing chemotherapy treatments. Additionally, greater sensitivity and quantity among plasma concentrations was exhibited by CT-proET-1, with an appropriate index to detect severity better than ELDP. Consideration of biomarker status for ELDP and CT-proET-1 is reliant upon verification of function.

ELDP was reported to function by enhancing vasoconstriction in ET-1-pretreated isolated resistance arteries [123]. However, treatment of mesenteric arteries with 10 nM ELDP alone produced no vasoconstrictive observation [123]. After pre-treatment with 1–3 nM of ET-1, ELDP treatment resulted in an enhanced vascular tone characterised by a 10 % increase in vasoconstriction [123]. Consistent with this observation, the duration of ET-1 (0.3 nmol/kg) response increased significantly when rats were administered with ELDP, compared to control [123]. Substantial differences were recorded at the 15-minute mark, showing a notably extended response (p < 0.02). In summary, the ratio of pre-peptide ELDP to ET-1 appears to play a significant role in its functionality, leading to an extended duration and an amplified pressor response. If this finding is validated, targeting ELDP for inhibition could potentially be a strategy to mitigate the vasoconstrictive potential of ET-1.

Table 3 summarises advantages and disadvantages of more stable biomarkers of ET-1 synthesis.

**Table 3:** Potential biomarkers for assessing endothelial dysfunction and their advantages and disadvantages.

Endothelial dysfunction biomarkers	Advantages	Disadvantages	Sources
Endothelin-1	- Incremental relationship towards poor prognosis. - Recognised vasopressor. - Repeated findings from modest and large cohort meta-analysis confirming role in pathogenesis. - Identified response to chemotherapy treatments.	- Short half-life. - Unclear clearance mechanism. - Rapid receptor uptake. - Unprovoked basolateral secretion. - Unstable in circulation.	[119]
Big-Endothelin-1	- Incremental relationship towards poor prognosis. - Recognised prohormone vasopressor.	- Shorter half-life in circulation compared to ET-1; 7.5 + 0.2 min ET-1, 3.9 + 0.2 min Big-ET-1. Although slower clearance via tissue distribution than ET-1, localised to hepatic and renal system. - Difficult to quantify in serum. - Hypersensitive, leading to false or misleading results.	[121,142]
Endothelin-Like Domain Peptide	- Longer half-life - More stable in circulation - Potentiates ET-1 activity - Good specification between low and high-risk diagnosis	- Little research available to fully scope potential and global influence upon the cardiovascular system. - Further investigation needed for clinical application for risk profiling.	[70,123,133]
CT-proET-1	- Longest half-life among proET-1 peptides. - Stable in circulation. - Assessed in multiple studies and demonstrated reproducibility. - Significant correlation for disease severity, with sensitive identification for vascular diseases.	- Unclear function and role within the vascular system. - Further analysis required for clinical application and risk profiling.	[70,123,133,138,[143], [144], [145], [146], [147], [148]]

## 6 CT-proET-1 as a potential biomarker for endothelial dysfunction

The physiological function of CT-proET-1 has yet to be determined, still consistent data has suggested CT-proET-1 as a potential predictor for systemic and cardiovascular diseases [70,138,143,145,149]. Exploring the identification of the CT-proET-1 ligand would enhance our understanding of the fundamental mechanisms and physiological impact of this peptide.

Serum levels of CT-proET-1 have been reported to be significantly greater in patients from systemic and cardiac illnesses, as expected due its precursor representation for ET-1 presence. A study by Papassotiriou et al. confirmed CT-proET-1 to be employed as an indirect measure of ET-1, with replicable correlative results of increased amounts from septic shock and congestive heart failure against controls (median 189 pmol/L and 104 pmol/L respectively) [143]. Results also indicated a 0.8 correlation for big-ET-1 for 43 subjects, (p = 0.0001) [143]. Analysis of renal failure and CKD has repeatedly established amplified CT-proET-1 serum values [123,146]. Similar findings were also observed in other studies regarding PAH. Marques et al. followed 28 patients for 12-months and monitored cardiovascular biomarkers for comparative investigation for risk stratification; markers included CT-proET-1 and previously described NT-proBNP and cTnI [144]. Association with increased hazard of reaching primary composite end-point was observed exclusively by CT-proET-1 (increase 10.1; 95 % CI 2.0–50.6). In conclusion, there is a notable association among the renal, pulmonary, and cardiac systems, facilitated by interchangeable markers capable of identifying HTN and cardiovascular damage. Larger cohort studies have conducted additional investigations into the potential of CT-proET-1 as a prognostic marker spanning various cardiovascular diseases and instances of organ failure.

Buendgens et al. investigated 217 patients following intensive care admissions, 144 with sepsis and 73 without [138]. Data showed enlarged quantities of CT-proET-1 in septic cases, with correlative indications to severity of disease for organ failure incorporating renal, hepatic, and cardiac dysfunction [138]. Patients were matched between the two diagnoses, septic or non-septic, and grouped based on sex, age, and disease severity. As understood, cardiometabolite diseases and inflammation increase vasopressor demand [150]; subanalysis of CT-proET-1 showed higher levels in septic groups (p < 0.001). A significant association of the current cardiac biomarker BNP was observed against CT-proET-1 (r = 0.505, p < 0.001) [138]. Individuals with greater CT-proET-1 serum levels at admission obtained higher risk of death while in intensive care (median 88.3 vs 58.2 pmol/L; p = 0.029) and within the 3 year overall mortality follow-up (median 89.9 vs 53.7 pmol/L; p = 0.003) [138]; thus, staging CT-proET-1 as a potential indicator for risk stratification.

This risk stratification has been observed in more recent studies to assess arrhythmic death in patients with known underlying heart diseases, HF and reduced LVEF. Burger et al. observed CT-proET-1 plasma levels among 160 HF patients over 7 years [147]. CT-proET-1 was shown to be associated with significantly higher risk of arrhythmic death or resuscitated cardiac arrest in ischaemic cardiomyopathy, hazard ratio: 95 % 1.07 (p = 0.045). Interestingly, another study found no predictive utility for identifying higher risk patients for non-ischaemic dilated cardiomyopathy [147]. This observation may suggest a characteristic related to perfusion, with potential feedback mechanisms triggered by inadequate oxygen supply that initiates vasoconstriction to meet metabolic demands. Additional evidence to support the hypoxic role hypothesis includes a 2019 study by Oers et al. which assessed inflammatory markers in 105 critically ill patients with SARS-Cov2 pneumonia and evaluated association with obesity [148]. Unlike other vasopressors and associated markers, CT-proET-1 did not display a correlation to obesity. This demonstrates CT-proET-1 as an independent variable for patients with poor perfusion and not for chronic inflammation caused by cardiometabolite or cardiovascular diseases.

The relationship between ischaemia and CT-proET-1 was moreover demonstrated with pathophysiology of ischaemic stroke. Westphal et al. conducted serial measurements of the precursor in 361 patients of ischaemic stroke, with assessed mortality after 90 days [145]. The univariate analysis determined unfavourable outcome for individuals with peaked concentrations from their initial sample following admission. Prognostic values included odd ratio of 1.32 (95 % CI, 1.16–1.51 p < 0.001) for unfavourable outcome, with adjustments CT-proET-1 remained an independent predictor for mortality and a hazard ratio of 1.50 (95 % CI, 1.29–1.74 p < 0.001) [145]. This study revealed CT-proET-1 to be a prospective prognostic marker for ischaemic related disease, with greater accuracy than existing biomarkers.

Despite lack of understanding of the functional aspects of CT-proET-1, the correlation of this precursor to predict prognosis has been repeatedly observed and could provision risk assessments for patients with underlying heart diseases prior to chemotherapy cycles. Future clinical routine could adopt CT-proET-1 peptide as a biomarker for treatment efficacy and patient care, with reduced plasma concentrations suggesting therapeutic benefits. Hypoxic mechanisms of CT-proET-1 could additionally be therapeutically manipulated to limit ischaemic damage. Further research is required to ascertain the molecular processes of CT-proET-1 for optimum endothelial function and cardiovascular health.

## 7 Conclusion

Endothelial dysfunction is a well-established phenomenon that leads to cardiovascular disease and injury to high-metabolic organs, including pulmonary and renal systems. Chemotherapy-induced cardiotoxicity remains a clinical challenge with and monitoring this is crucial in detection. Although increased levels of established biomarkers, cardiac troponin, BNP, or NT-proBNP frequently correlate with reductions in LVEF, playing an important role in the prediction of cardiovascular toxicity, there is still a need for biomarkers providing early identification of cardiotoxicity. Thus, a consensus is still not achieved on the use of biomarkers by the international guidelines.

Biomarkers for endothelial dysfunction include the potent vasopressor peptide ET-1. There is even a greater potential for detection with the co-synthesised peptide fragments: ELDP and CT-proET-1 with favourable stability in the circulatory system. ELDP demonstrated physiological influence upon ET-1, with enhanced vasoconstrictive function and prolonged duration of action. ELDP therefore has a substantial credit of being a physiologically relevant biomarker. On the other hand, CT-proET-1 having longest half-life among the ET-1 peptide fragments could confer an advantage of stability, enabling better risk-stratification during cardiotoxicity monitoring. Novel insight towards ELDP and CT-proET-1 could provide opportunity for clinical trials regarding ET-1 modulation and risk stratification, respectively. Neither are currently undergoing clinical trials.

Further research is needed with these endothelial biomarkers:

(i) During the course of chemotherapy using larger sample size or large clinical trials.
(ii) To test their sensitivity in different forms of cardiotoxicity that may be caused by different cancer treatments.
(iii) To compare these with the most strongly proposed blood biomarkers (troponin or NT-proBNP) and cardiac imaging modalities; previously, additional benefit of combining blood-based biomarkers with cardiac imaging was shown to identify cardiotoxicity risk.
(iv) To compare their benefit when used in combination with other biomarkers for risk-stratification. Multimarker strategy could add prognostic value enabling earlier identification.
(v) Aiding help from machine learning based methodologies could add further value in decision-making or risk-stratification processes to decide on the most promising combinations.

Some additional investigation would be also valuable for the endothelial research to understand binding mechanism between ET-1 and ELDP that supports the vasopressor response that may be a novel target.

In conclusion, these stable peptide fragments of ET-1 synthesis are promising biomarkers. Elevations are indicative of endothelial dysfunction and may confer added value for identifying high-risk patients earlier. Further studies are required to clarify their role in risk-stratification of chemotherapy-induced cardiotoxicity.

## Ethics approval and consent to participate

Not applicable.

## Consent for publication

Not applicable.

## Funding

Not funded externally.

## CRediT authorship contribution statement

**Gabrielle Boutin:** Writing – original draft. **Jale Yuzugulen:** Writing – review & editing. **Md Zahidul Islam Pranjol:** Writing – review & editing, Supervision, Conceptualization.

## Declaration of competing interest

The authors declare that they have no competing interests.

## Data Availability

Not applicable.

## References

[1] Herrmann J. (2020). Adverse cardiac effects of cancer therapies: cardiotoxicity and arrhythmia. Nat Rev Cardiol.

[2] Tian C., Yang Y., Bai B., Wang S., Liu M., Sun R.-C. (2021). Potential of exosomes as diagnostic biomarkers and therapeutic carriers for doxorubicin-induced cardiotoxicity. Int J Biol Sci.

[3] Mclaughlin M., Florida-James G., Ross M. (2021). Breast cancer chemotherapy vascular toxicity: a review of mediating mechanisms and exercise as a potential therapeutic. Vasc Biol.

[4] Oeffinger K.C., Mertens A.C., Sklar C.A., Kawashima T., Hudson M.M., Meadows A.T. (2006). Chronic health conditions in adult survivors of childhood cancer. N Engl J Med.

[5] Patnaik J.L., Byers T., DiGuiseppi C., Dabelea D., Denberg T.D. (2011). Cardiovascular disease competes with breast cancer as the leading cause of death for older females diagnosed with breast cancer: a retrospective cohort study. Breast Cancer Res.

[6] Cardinale D., Iacopo F., Cipolla C.M. (2020). Cardiotoxicity of anthracyclines. Front Cardiovasc Med.

[7] Bansal N., Amdani S., Lipshultz E.R., Lipshultz S.E. (2017). Chemotherapy-induced cardiotoxicity in children. Expert Opin Drug Metab Toxicol.

[8] Campia U., Moslehi J.J., Amiri-Kordestani L., Barac A., Beckman J.A., Chism D.D. (2019). Cardio-oncology: vascular and metabolic perspectives: a scientific statement from the American Heart Association. Circulation..

[9] Bellinger A.M., Arteaga C.L., Force T., Humphreys B.D., Demetri G.D., Druker B.J. (2015). Cardio-oncology: how new targeted cancer therapies and precision medicine can inform cardiovascular discovery. Circulation..

[10] Christenson E.S., James T., Agrawal V., Park B.H. (2015). Use of biomarkers for the assessment of chemotherapy-induced cardiac toxicity. Clin Biochem.

[11] Semeraro G.C., Cipolla C.M., Cardinale D.M. (2021). Role of cardiac biomarkers in cancer patients. Cancers.

[12] Jin J.-f., Zhu L.-l., Chen M., Xu H.-m., Wang H.-f., Feng X.-q. (2015). The optimal choice of medication administration route regarding intravenous, intramuscular, and subcutaneous injection. Patient Prefer Adherence.

[13] Abraham G.R., Davenport A.P. (2023). From ABCD to E for endothelin in resistant hypertension. Cell.

[14] Davenport A.P., Hyndman K.A., Dhaun N., Southan C., Kohan D.E., Pollock J.S. (2016). Endothelin. Pharmacol Rev.

[15] Herrmann J. (2020). Vascular toxic effects of cancer therapies. Nat Rev Cardiol.

[16] Molema G., Zijlstra J.G., van Meurs M., Kamps J.A. (2022). Renal microvascular endothelial cell responses in sepsis-induced acute kidney injury. Nat Rev Nephrol.

[17] Hader S.N., Zinkevich N., Norwood Toro L.E., Kriegel A.J., Kong A., Freed J.K. (2019). Detrimental effects of chemotherapy on human coronary microvascular function. Am J Phys Heart Circ Phys.

[18] Avagimyan A. (2022). Hyperhomocysteinemia as a link of chemotherapy-related endothelium impairment. Curr Probl Cardiol.

[19] Terwoord J.D., Beyer A.M., Gutterman D.D. (2022). Endothelial dysfunction as a complication of anti-cancer therapy. Pharmacol Ther.

[20] Weir M.R. (2010). Endothelin-receptor antagonists for treating hypertension. Nat Rev Nephrol.

[21] Jin Q., Su H., Yang R., Tan Y., Li B., Yi W. (2021). C1q/TNF-related protein-9 ameliorates hypoxia-induced pulmonary hypertension by regulating secretion of endothelin-1 and nitric oxide mediated by AMPK in rats. Sci Rep.

[22] Mazur M., Wang F., Hodge D.O., Siontis B.L., Beinborn D.S., Villarraga H.R. (2017). Burden of cardiac arrhythmias in patients with anthracycline-related cardiomyopathy. JACC: Clin Electrophysiol.

[23] Demissei B.G., Hubbard R.A., Zhang L., Smith A.M., Sheline K., McDonald C. (2020). Changes in cardiovascular biomarkers with breast cancer therapy and associations with cardiac dysfunction. J Am Heart Assoc.

[24] Madeddu C., Deidda M., Piras A., Cadeddu C., Demurtas L., Puzzoni M. (2016). Pathophysiology of cardiotoxicity induced by nonanthracycline chemotherapy. J Cardiovasc Med.

[25] Mudd T.W., Khalid M., Guddati A.K. (2021). Cardiotoxicity of chemotherapy and targeted agents. Am J Cancer Res.

[26] Cai F., Luis M.A.F., Lin X., Wang M., Cai L., Cen C. (2019). Anthracycline-induced cardiotoxicity in the chemotherapy treatment of breast cancer: preventive strategies and treatment. Mol Clin Oncol.

[27] Chianca M., Fabiani I., Del Franco A., Grigoratos C., Aimo A., Panichella G. (2022). Management and treatment of cardiotoxicity due to anticancer drugs: 10 questions and answers. Eur J Prev Cardiol.

[28] Pai V.B., Nahata M.C. (2000). Cardiotoxicity of chemotherapeutic agents: incidence, treatment and prevention. Drug Saf.

[29] Radulescu L.M., Radulescu D., Ciuleanu T.-E., Crisan D., Buzdugan E., Romitan D.-M. (2021). Cardiotoxicity associated with chemotherapy used in gastrointestinal tumours. Medicina.

[30] Yu L.-R., Cao Z., Makhoul I., Daniels J.R., Klimberg S., Wei J.Y. (2018). Immune response proteins as predictive biomarkers of doxorubicin-induced cardiotoxicity in breast cancer patients. Exp Biol Med.

[31] Avila M.S., Siqueira S.R.R., Ferreira S.M.A., Bocchi E.A. (2019). Prevention and treatment of chemotherapy-induced cardiotoxicity. Methodist Debakey Cardiovasc J.

[32] Bloom M.W., Hamo C.E., Cardinale D., Ky B., Nohria A., Baer L. (2016). Cancer therapy–related cardiac dysfunction and heart failure: part 1: definitions, pathophysiology, risk factors, and imaging. Circ Heart Fail.

[33] Yeh E.T., Bickford C.L. (2009). Cardiovascular complications of cancer therapy: incidence, pathogenesis, diagnosis, and management. J Am Coll Cardiol.

[34] Ahlmann M., Hempel G. (2016). The effect of cyclophosphamide on the immune system: implications for clinical cancer therapy. Cancer Chemother Pharmacol.

[35] Martin M., Fornecker L., Marcellin L., Mousseaux E., Hij A., Snowden J. (2017). Acute and fatal cardiotoxicity following high-dose cyclophosphamide in a patient undergoing autologous stem cell transplantation for systemic sclerosis despite satisfactory cardiopulmonary screening. Bone Marrow Transplant.

[36] Bowles E.J.A., Wellman R., Feigelson H.S., Onitilo A.A., Freedman A.N., Delate T. (2012). Risk of heart failure in breast cancer patients after anthracycline and trastuzumab treatment: a retrospective cohort study. J Natl Cancer Inst.

[37] Kong C.-Y., Guo Z., Song P., Zhang X., Yuan Y.-P., Teng T. (2022). Underlying the mechanisms of doxorubicin-induced acute cardiotoxicity: oxidative stress and cell death. Int J Biol Sci.

[38] Shelburne N., Simonds N.I., Adhikari B., Alley M., Desvigne-Nickens P., Dimond E. (2019). Changing hearts and minds: improving outcomes in cancer treatment-related cardiotoxicity. Curr Oncol Rep.

[39] Thomas S.A. (2017). Chemotherapy agents that cause cardiotoxicity. US Pharm.

[40] Cardinale D., Colombo A., Bacchiani G., Tedeschi I., Meroni C.A., Veglia F. (2015). Early detection of anthracycline cardiotoxicity and improvement with heart failure therapy. Circulation..

[41] Armenian S.H., Lacchetti C., Barac A., Carver J., Constine L.S., Denduluri N. (2017). Prevention and monitoring of cardiac dysfunction in survivors of adult cancers: American Society of Clinical Oncology Clinical Practice Guideline. J Clin Oncol.

[42] Henriksen P.A. (2018). Anthracycline cardiotoxicity: an update on mechanisms, monitoring and prevention. Heart..

[43] Shandilya M., Sharma S., Das P.P., Charak S. (2020). Advances in precision medicine oncology.

[44] Jacobse J.N., Steggink L.C., Sonke G.S., Schaapveld M., Hummel Y.M., Steenbruggen T.G. (2020). Myocardial dysfunction in long-term breast cancer survivors treated at ages 40–50 years. Eur J Heart Fail.

[45] Jacobse J.N., Schaapveld M., Boekel N.B., Hooning M.J., Jager A., Baaijens M.H. (2021). Risk of heart failure after systemic treatment for early breast cancer: results of a cohort study. Breast Cancer Res Treat.

[46] Triulzi T., Forte L., Regondi V., Di Modica M., Ghirelli C., Carcangiu M.L. (2019). HER2 signaling regulates the tumor immune microenvironment and trastuzumab efficacy. Oncoimmunology..

[47] Cameron D., Piccart-Gebhart M.J., Gelber R.D., Procter M., Goldhirsch A., de Azambuja E. (2017). 11 years’ follow-up of trastuzumab after adjuvant chemotherapy in HER2-positive early breast cancer: final analysis of the HERceptin Adjuvant (HERA) trial. Lancet.

[48] Wang F., Chandra J., Kleinerman E.S. (2021). Exercise intervention decreases acute and late doxorubicin-induced cardiotoxicity. Cancer Med.

[49] Chang V.Y., Wang J.J. (2018). Pharmacogenetics of chemotherapy-induced cardiotoxicity. Curr Oncol Rep.

[50] Banke A., Fosbøl E.L., Ewertz M., Videbæk L., Dahl J.S., Poulsen M.K. (2019). Long-term risk of heart failure in breast cancer patients after adjuvant chemotherapy with or without trastuzumab. JACC Heart Fail.

[51] Rehman F.U., Al-Waeel M., Naz S.S., Shah K.U. (2020). Anticancer therapeutics: a brief account on wide refinements. Am J Cancer Res.

[52] Duarte J.A., Gomes E.R., De Barros A.L.B., Leite E.A. (2023). Co-encapsulation of simvastatin and doxorubicin into pH-sensitive liposomes enhances antitumoral activity in breast cancer cell lines. Pharmaceutics..

[53] Nel J., Elkhoury K., Velot É., Bianchi A., Acherar S., Francius G. (2023). Functionalized liposomes for targeted breast cancer drug delivery. Bioact Mater.

[54] Song D., Shabani J., Jaiswal V., Cagliostro M., Rubinstein D., Alraies M.C. (2023). Delayed doxorubicin induced cardiomyopathy in a breast cancer patient: a case report. Radiol Case Rep.

[55] Nonaka M., Hosoda H., Uezono Y. (2021). Cancer treatment-related cardiovascular disease: current status and future research priorities. Biochem Pharmacol.

[56] Gai W., An J., Wang Z., Han X., Geng J., Liang Y. (2021). Research progress of biomarkers in early detection of chemotherapy-induced cardiotoxicity. Heart Fail Rev.

[57] Stansfeld A., Radia U., Goggin C., Mahalingam P., Benson C., Napolitano A. (2022). Pharmacological strategies to reduce anthracycline-associated cardiotoxicity in cancer patients. Expert Opin Pharmacother.

[58] Goutsouliak K., Veeraraghavan J., Sethunath V., De Angelis C., Osborne C.K., Rimawi M.F. (2020). Towards personalized treatment for early stage HER2-positive breast cancer. Nat Rev Clin Oncol.

[59] de Wall C., Bauersachs J., Berliner D. (2021). Cardiooncology—dealing with modern drug treatment, long-term complications, and cancer survivorship. Clin Exp Metastasis.

[60] Arnouk H., Hassan B. (2021).

[61] Sobczuk P., Czerwińska M., Kleibert M., Cudnoch-Jędrzejewska A. (2022). Anthracycline-induced cardiotoxicity and renin-angiotensin-aldosterone system—from molecular mechanisms to therapeutic applications. Heart Fail Rev.

[62] Rajendran P., Rengarajan T., Thangavel J., Nishigaki Y., Sakthisekaran D., Sethi G. (2013). The vascular endothelium and human diseases. Int J Biol Sci.

[63] Todorova V.K., Hsu P.C., Wei J.Y., Lopez-Candales A., Chen J.Z., Su L.J. (2020). Biomarkers of inflammation, hypercoagulability and endothelial injury predict early asymptomatic doxorubicin-induced cardiotoxicity in breast cancer patients. Am J Cancer Res.

[64] Haynes W.G., Webb D.J. (1998). Endothelin as a regulator of cardiovascular function in health and disease. J Hypertens.

[65] Masaki T., Sawamura T. (2006). Endothelin and endothelial dysfunction. Proc Jpn Acad Ser B Phys Biol Sci.

[66] Macedo A.V.S., de Carvalho e Silva C.M.P.D., Pellegrino L.B., PTF Marcatti, Zerati A.E., Nishinari K., Wolosker N. (2022). Vascular surgery in oncology.

[67] Chong J.H., Ghosh A.K. (2019). Coronary artery vasospasm induced by 5-fluorouracil: proposed mechanisms, existing management options and future directions. Interv Cardiol.

[68] Wu Q., Bai B., Tian C., Li D., Yu H., Song B. (2022). The molecular mechanisms of cardiotoxicity induced by HER2, VEGF, and tyrosine kinase inhibitors: an updated review. Cardiovasc Drugs Ther.

[69] Abdul-Rahman T., Dunham A., Huang H., Bukhari S.M.A., Mehta A., Awuah W.A. (2023). Chemotherapy induced cardiotoxicity: a state of the art review on general mechanisms, prevention, treatment and recent advances in novel therapeutics. Curr Probl Cardiol.

[70] Dhaun N., Yuzugulen J., Kimmitt R.A., Wood E.G., Chariyavilaskul P., MacIntyre I.M. (2015). Plasma pro-endothelin-1 peptide concentrations rise in chronic kidney disease and following selective endothelin a receptor antagonism. J Am Heart Assoc.

[71] Brandes R.P. (2014). Endothelial dysfunction and hypertension. J Am Heart Assoc Hypertens.

[72] Soultati A., Mountzios G., Avgerinou C., Papaxoinis G., Pectasides D., Dimopoulos M.-A. (2012). Endothelial vascular toxicity from chemotherapeutic agents: preclinical evidence and clinical implications. Cancer Treat Rev.

[73] Wilson C., Zhang X., Buckley C., Heathcote H.R., Lee M.D., McCarron J. (2019). Increased vascular contractility in hypertension results from impaired endothelial calcium signaling. Journal of American Heart Association Hypertension..

[74] Saxena T., Ali A.O., Saxena M. (2018). Pathophysiology of essential hypertension: an update. Expert Rev Cardiovasc Ther.

[75] Porter C., Azam T.U., Mohananey D., Kumar R., Chu J., Lenihan D. (2022). Permissive cardiotoxicity. JACC: CardioOncology.

[76] Thygesen K., Alpert J.S., Jaffe A.S., Chaitman B.R., Bax J.J., Morrow D.A. (2018). Fourth universal definition of myocardial infarction (2018). Circulation..

[77] Hamm C.W., Goldmann B.U., Heeschen C., Kreymann G., Berger J., Meinertz T. (1997). Emergency room triage of patients with acute chest pain by means of rapid testing for cardiac troponin T or troponin I. N Engl J Med.

[78] Damman P., van’t Hof A., Ten Berg J., Jukema J., Appelman Y., Liem A. (2015). ESC guidelines for the management of acute coronary syndromes in patients presenting without persistent ST-segment elevation: comments from the Dutch ACS working group. Neth Hear J.

[79] Bhagat A., Kleinerman E.S. (2020). Current advances in osteosarcoma.

[80] McGowan J.V., Chung R., Maulik A., Piotrowska I., Walker J.M., Yellon D.M. (2017). Anthracycline chemotherapy and cardiotoxicity. Cardiovasc Drugs Ther.

[81] Lakhani H.V., Pillai S.S., Zehra M., Dao B., Tirona M.T., Thompson E. (2021). Detecting early onset of anthracyclines-induced cardiotoxicity using a novel panel of biomarkers in West-Virginian population with breast cancer. Sci Rep.

[82] Ky B., Putt M., Sawaya H., French B., Januzzi J.L., Sebag I.A. (2014). Early increases in multiple biomarkers predict subsequent cardiotoxicity in patients with breast cancer treated with doxorubicin, taxanes, and trastuzumab. J Am Coll Cardiol.

[83] Michel L., Mincu R.I., Mrotzek S.M., Korste S., Neudorf U., Rassaf T. (2020). Cardiac biomarkers for the detection of cardiotoxicity in childhood cancer—a meta-analysis. ESC Heart Fail.

[84] Cardinale D., Sandri M.T., Colombo A., Colombo N., Boeri M., Lamantia G. (2004). Prognostic value of troponin I in cardiac risk stratification of cancer patients undergoing high-dose chemotherapy. Circulation..

[85] Wu A.H. (2017). Release of cardiac troponin from healthy and damaged myocardium. Front Lab Med.

[86] Burridge P.W., Li Y.F., Matsa E., Wu H., Ong S.-G., Sharma A. (2016). Human induced pluripotent stem cell–derived cardiomyocytes recapitulate the predilection of breast cancer patients to doxorubicin-induced cardiotoxicity. Nat Med.

[87] Sawaya H., Sebag I.A., Plana J.C., Januzzi J.L., Ky B., Tan T.C. (2012). Assessment of echocardiography and biomarkers for the extended prediction of cardiotoxicity in patients treated with anthracyclines, taxanes, and trastuzumab. Circ Cardiovasc Imaging.

[88] Kuwahara K. (2021). The natriuretic peptide system in heart failure: diagnostic and therapeutic implications. Pharmacol Ther.

[89] Brady B., King G., Murphy R.T., Walsh D. (2022). Myocardial strain: a clinical review. Ir J Med Sci.

[90] Tan L.-L., Lyon A.R. (2018). Role of biomarkers in prediction of cardiotoxicity during cancer treatment. Curr Treat Options Cardiovasc Med.

[91] Ananthan K., Lyon A.R. (2020). The role of biomarkers in cardio-oncology. J Cardiovasc Transl Res.

[92] Yancy C.W., Jessup M., Bozkurt B., Butler J., Casey D.E., Drazner M.H. (2013). 2013 ACCF/AHA guideline for the management of heart failure: a report of the American College of Cardiology Foundation/American Heart Association Task Force on Practice Guidelines. J Am Coll Cardiol.

[93] Rüger A.M., Schneeweiss A., Seiler S., Tesch H., van Mackelenbergh M., Marmé F. (2020). Cardiotoxicity and cardiovascular biomarkers in patients with breast cancer: data from the GeparOcto-GBG 84 trial. J Am Heart Assoc.

[94] Lenihan D., Stevens P., Massey M., Plana J., Araujo D., Fanale M. (2016). The utility of point-of-care biomarkers to detect cardiotoxicity during anthracycline chemotherapy: a feasibility study. J Card Fail.

[95] Hinton J., Gabara L., Curzen N. (2020). Is the true clinical value of high-sensitivity troponins as a biomarker of risk? The concept that detection of high-sensitivity troponin ‘never means nothing’. Expert Rev Cardiovasc Ther.

[96] Januzzi J.L., Mahler S.A., Christenson R.H., Rymer J., Newby L.K., Body R. (2019). Recommendations for institutions transitioning to high-sensitivity troponin testing. J Am Coll Cardiol.

[97] Nellipudi J.A., Baker R.A., Dykes L., Krieg B.M., Bennetts J.S. (2021). Prognostic value of high-sensitivity troponin T after on-pump coronary artery bypass graft surgery. Heart Lung Circ.

[98] Goryacheva O.A., Ponomaryova T.D., Drozd D.D., Kokorina A.A., Rusanova T.Y., Mishra P.K. (2022). Heart failure biomarkers BNP and NT-proBNP detection using optical labels. TrAC Trends Anal Chem.

[99] Prontera C., Zucchelli G.C., Vittorini S., Storti S., Emdin M., Clerico A. (2009). Comparison between analytical performances of polyclonal and monoclonal electrochemiluminescence immunoassays for NT-proBNP. Clin Chim Acta.

[100] Castiglione V., Aimo A., Vergaro G., Saccaro L., Passino C., Emdin M. (2022). Biomarkers for the diagnosis and management of heart failure. Heart Fail Rev.

[101] Krim S.R., Vivo R.P., Krim N.R., Qian F., Cox M., Ventura H. (2013). Racial/ethnic differences in B-type natriuretic peptide levels and their association with care and outcomes among patients hospitalized with heart failure: findings from Get With The Guidelines-Heart Failure. JACC Heart Fail.

[102] Bozkurt B., Coats A.J.S., Tsutsui H., Abdelhamid C.M., Adamopoulos S., Albert N. (2021). Universal definition and classification of heart failure: a report of the Heart Failure Society of America, Heart Failure Association of the European Society of Cardiology, Japanese Heart Failure Society and Writing Committee of the Universal Definition of Heart Failure: Endorsed by the Canadian Heart Failure Society, Heart Failure Association of India, Cardiac Society of Australia and New Zealand, and Chinese Heart Failure Association. Eur J Heart Fail.

[103] McDonagh T.A., Metra M., Adamo M., Gardner R.S., Baumbach A., Bohm M. (2021). 2021 ESC guidelines for the diagnosis and treatment of acute and chronic heart failure. Eur Heart J.

[104] Nguyen K., Fan W., Bertoni A., Budoff M.J., Defilippi C., Lombardo D. (2020). N-terminal pro B-type natriuretic peptide and high-sensitivity cardiac troponin as markers for heart failure and cardiovascular disease risks according to glucose status (from the Multi-Ethnic Study of Atherosclerosis [MESA]). Am J Cardiol.

[105] Kruger S., Hoffmann R., Graf Jr, Janssens U., Hanrath P. (2003). Brain natriuretic peptide. Med Klin.

[106] Welsh P., Campbell R.T., Mooney L., Kimenai D.M., Hayward C., Campbell A. (2022). Reference ranges for NT-proBNP (N-terminal pro-B-type natriuretic peptide) and risk factors for higher NT-proBNP concentrations in a large general population cohort. Circ Heart Fail.

[107] Hildebrandt P., Collinson P.O., Doughty R.N., Fuat A., Gaze D.C., Gustafsson F. (2010). Age-dependent values of N-terminal pro-B-type natriuretic peptide are superior to a single cut-point for ruling out suspected systolic dysfunction in primary care. Eur Heart J.

[108] Radha R., Shahzadi S.K., Al-Sayah M.H. (2021). Fluorescent immunoassays for detection and quantification of cardiac troponin I: a short review. Molecules..

[109] Zhao H., Su E., Huang L., Zai Y., Liu Y., Chen Z. (2022). Washing-free chemiluminescence immunoassay for rapid detection of cardiac troponin I in whole blood samples. Chin Chem Lett.

[110] Wang Y., Yang Y., Chen C., Wang S., Wang H., Jing W. (2020). One-step digital immunoassay for rapid and sensitive detection of cardiac troponin I. ACS Sens.

[111] Fellner S., Hentze S., Kempin U., Richter E., Rocktäschel J., Langer B. (2015). Analytical evaluation of a BNP assay on the new point-of-care platform respons®IQ. Pract Lab Med.

[112] Abdel Ghafar M.T. (2020). An overview of the classical and tissue-derived renin-angiotensin-aldosterone system and its genetic polymorphisms in essential hypertension. Steroids..

[113] Aroor A., DeMarco V., Jia G., Sun Z., Nistala R., Meininger G. (2013). The role of tissue renin-angiotensin-aldosterone system in the development of endothelial dysfunction and arterial stiffness. Front Endocrinol.

[114] Yanagisawa M.B.M. (2019). Endothelin: 30 years from discovery to therapy. J Am Heart Assoc Hypertens.

[115] Dmour B.A., Costache A.D., Dmour A., Huzum B., Duca S.T., Chetran A. (2023). Could endothelin-1 be a promising neurohormonal biomarker in acute heart failure?. Diagnostics.

[116] Zymlinski R., Sokolski M., Biegus J., Siwolowski P., Nawrocka-Millward S., Sokolska J.M. (2019). Multi-organ dysfunction/injury on admission identifies acute heart failure patients at high risk of poor outcome. Eur J Heart Fail.

[117] Yeoh S.E., Docherty K.F., Campbell R.T., Jhund P.S., Hammarstedt A., Heerspink H.J.L. (2023). Endothelin-1, outcomes in patients with heart failure and reduced ejection fraction, and effects of dapagliflozin: findings from DAPA-HF. Circulation..

[118] Jankowich M., Choudhary G. (2020). Endothelin-1 levels and cardiovascular events. Trends Cardiovasc Med.

[119] Krishnarao K., Bruno K.A., Di Florio D.N., Edenfield B.H., Whelan E.R., Macomb L.P. (2022). Upregulation of Endothelin-1 may predict chemotherapy-induced cardiotoxicity in women with breast cancer. J Clin Med.

[120] Zhang C., Tian J., Jiang L., Xu L., Liu J., Zhao X. (2019). Prognostic value of plasma big Endothelin-1 level among patients with three-vessel disease: a cohort study. J Atheroscler Thromb.

[121] Zhou B.-Y., Guo Y.-L., Wu N.-Q., Zhu C.-G., Gao Y., Qing P. (2017). Plasma big endothelin-1 levels at admission and future cardiovascular outcomes: a cohort study in patients with stable coronary artery disease. Int J Cardiol.

[122] Kowalczyk A., Kleniewska P., Kolodziejczyk M., Skibska B., Goraca A. (2015). The role of endothelin-1 and endothelin receptor antagonists in inflammatory response and sepsis. Arch Immunol Ther Exp.

[123] Yuzugulen J., Douthwaite J.A., Wood E.G., Villar I.C., Patel N.S.A., Jegard J. (2017). Characterisation of preproendothelin-1 derived peptides identifies endothelin-like domain peptide as a modulator of Endothelin-1. Sci Rep.

[124] Gasic S., Wagner O., Vierhapper H., Nowotny P., Waldhäusl W. (1992). Regional hemodynamic effects and clearance of endothelin-1 in humans: renal and peripheral tissues may contribute to the overall disposal of the peptide. J Cardiovasc Pharmacol.

[125] Inoue A., Yanagisawa M., Takuwa Y., Mitsui Y., Kobayashi M., Masaki T. (1989). The human preproendothelin-1 gene: complete nucleotide sequence and regulation of expression. J Biol Chem.

[126] Moon D.G., Horgan M.J., Andersen T.T., Krystek S.R., Fenton J.W., Malik A.B. (1989). Endothelin-like pulmonary vasoconstrictor peptide release by alpha-thrombin. Proc Natl Acad Sci.

[127] Turner A.J., Murphy L.J. (1996). Molecular pharmacology of endothelin converting enzymes. Biochem Pharmacol.

[128] Houde M., Desbiens L., D’Orléans-Juste P. (2016). Endothelin-1: biosynthesis, signaling and vasoreactivity. Adv Pharmacol.

[129] Yang L.L., Gros R., Kabir M.G., Sadi A., Gotlieb A.I., Husain M. (2004). Conditional cardiac overexpression of endothelin-1 induces inflammation and dilated cardiomyopathy in mice. Circulation..

[130] Struck J., Morgenthaler N.G., Bergmann A. (2005). Proteolytic processing pattern of the endothelin-1 precursor in vivo. Peptides..

[131] (2010). Biomarkers on a roll. Nat Biotechnol.

[132] Yuzugulen J., Lilitkarntakul P., Wood E.G., Kimmitt R.A., Dhaun N., Goddard J.G. (2013). Evaluation of urinary and plasma endothelin-like domain peptide (ELDP) in chronic kidney disease. Life Sci.

[133] Mullens W., Damman K., Testani J.M., Martens P., Mueller C., Lassus J. (2020). Evaluation of kidney function throughout the heart failure trajectory–a position statement from the Heart Failure Association of the European Society of Cardiology. Eur J Heart Fail.

[134] Marti C.N., Gheorghiade M., Kalogeropoulos A.P., Georgiopoulou V.V., Quyyumi A.A., Butler J. (2012). Endothelial dysfunction, arterial stiffness, and heart failure. J Am Coll Cardiol.

[135] Canaud B., Stephens M.P., Nikam M., Etter M., Collins A. (2021). Multitargeted interventions to reduce dialysis-induced systemic stress. Clin Kidney J.

[136] Ranchoux B., Günther S., Quarck R., Chaumais M.-C., Dorfmüller P., Antigny F. (2015). Chemotherapy-induced pulmonary hypertension: role of alkylating agents. Am J Pathol.

[137] Sellmer A., Hjortdal V.E., Bjerre J.V., Schmidt M.R., Bech B.H., Henriksen T.B. (2020). Cardiovascular biomarkers in the evaluation of patent ductus arteriosus in very preterm neonates: a cohort study. Early Hum Dev.

[138] Buendgens L., Yagmur E., Bruensing J., Herbers U., Baeck C., Trautwein C. (2017). C-terminal proendothelin-1 (CT-proET-1) is associated with organ failure and predicts mortality in critically ill patients. J Intensive Care.

[139] Katsurada K., Shinohara K., Aoki J., Nanto S., Kario K. (2022). Renal denervation: basic and clinical evidence. Hypertens Res.

[140] Rajagopal S., Yen-Rei A.Y. (2022). The pathobiology of pulmonary arterial hypertension. Cardiol Clin.

[141] Szczepaniak P., Siedlinski M., Hodorowicz-Zaniewska D., Nosalski R., Mikolajczyk T.P., Dobosz A.M. (2022). Breast cancer chemotherapy induces vascular dysfunction and hypertension through a NOX4-dependent mechanism. J Clin Invest.

[142] Burkhardt M., Barton M., Shaw S.G. (2000). Receptor- and non-receptor-mediated clearance of big-endothelin and endothelin-1: differential effects of acute and chronic ETA receptor blockade. J Hypertens.

[143] Papassotiriou J., Morgenthaler N.G., Struck J., Alonso C., Bergmann A. (2006). Immunoluminometric assay for measurement of the C-terminal endothelin-1 precursor fragment in human plasma. Clin Chem.

[144] Silva Marques J., Martins S.R., Calisto C., Gonçalves S., Almeida A.G., de Sousa J.C. (2013). An exploratory panel of biomarkers for risk prediction in pulmonary hypertension: emerging role of CT-proET-1. J Heart Lung Transplant.

[145] Westphal L.P., Schweizer J., Fluri F., De Marchis G.M., Christ-Crain M., Luft A.R. (2021). C-terminal-pro-endothelin-1 adds incremental prognostic value for risk stratification after ischemic stroke. Front Neurol.

[146] Dhaun N., Webb D.J. (2019). Endothelins in cardiovascular biology and therapeutics. Nat Rev Cardiol.

[147] Burger A., Stojkovic S., Diedrich A., Wojta J., Demyanets S., Pezawas T. (2021). Cardiac biomarkers for risk stratification of arrhythmic death in patients with heart failure and reduced ejection fraction. Br J Biomed Sci.

[148] van Oers J.A.H., Pouwels S., Ramnarain D., Kluiters Y., Bons J.A.P., de Lange D.W. (2022). Mid-regional proadrenomedullin, C-terminal proendothelin-1 values, and disease course are not different in critically ill SARS-CoV-2 pneumonia patients with obesity. Int J Obes.

[149] Gerull R., Neumann R.P., Atkinson A., Bernasconi L., Schulzke S.M., Wellmann S. (2022). Respiratory morbidity in preterm infants predicted by natriuretic peptide (MR-proANP) and endothelin-1 (CT-proET-1). Pediatr Res.

[150] Boutagy N.E., Singh A.K., Sessa W.C. (2022). Targeting the vasculature in cardiometabolic disease. J Clin Invest.

